# Core atoms escape from the shell: reverse segregation of Pb–Al core–shell nanoclusters via nanoscale melting

**DOI:** 10.1186/s11671-023-03924-3

**Published:** 2023-11-17

**Authors:** Wenkai Wu, Theodoros Pavloudis, Richard E. Palmer

**Affiliations:** 1 Nanomaterials Lab, Mechanical Engineering, Swansea University, Bay Campus, Fabian Way, Swansea, SA1 8EN UK; 2 School of Physics, Faculty of Sciences, Aristotle University of Thessaloniki, 54124 Thessaloniki, Greece

**Keywords:** Core–shell, Nanoclusters, Nanoparticles, Melting, Molecular dynamics

## Abstract

Melting is a phase transition that profoundly affects the fabrication and diverse applications of metal nanoclusters. Core–shell clusters offer distinctive properties and thus opportunities compared with other classes of nano-alloys. Molecular dynamics simulations have been employed to investigate the melting behaviour of Pb–Al core–shell clusters containing a fixed Pb_147_ core and varying shell thickness. Our results show that the core and shell melt separately. Surprisingly, core melting always drives the core Pb atoms to break out the shell and coat the nanoclusters in a reversed segregation process at the nanoscale. The melting point of the core increases with the shell thickness to exceed that of the bare core cluster, but the thinnest shell always supresses the core melting point. These results can be a reference for the future fabrication, manipulation, and exploitation of the core–shell nanoalloys chosen. The system chosen is ideally suited for experimental observations.

**Supplementary Information:**

The online version contains supplementary material available at 10.1186/s11671-023-03924-3.

## Introduction

Metal clusters have remarkable properties and numerous potential applications in catalysis, sensors, optoelectronics, biomedicine [1–5]. Critical factors which determine the properties of the clusters are their composition, size, and structure. In the case of alloy nanoclusters, the distribution of the different atoms inside the cluster. By tuning these factors, clusters with customized properties in principle can be fabricated [6–8]. In order, to realize such an approach, however, an extensive understanding of the properties of the clusters is essential. One of the most critical properties is the melting behaviour, which can have a crucial influence on the fabrication process as well as the application of these materials in real operational conditions [9].

Metal clusters generally exhibit a much reduced melting point than the corresponding bulk metals due to their enhanced surface-to-volume ratio. The depression of the melting point with decreasing size follows a scaling law [9] and has been predicted theoretically and reported experimentally for various metal clusters [9–11]. However, several exceptions have been found, including Sn [12, 13] and Ga [14]; the melting point of nanoclusters comprising these metals is much higher than that of the bulk materials when the cluster size is less than 40 atoms. This is attributed to the unique geometry of these clusters [13]. Meanwhile, it has also been shown that the melting point in some systems does not necessarily vary monotonically with the size, due to structural effects and competition between premelting and melting [15–17].

In experiments, the melting of large clusters is usually measured by electron or X-ray diffraction or nanocalorimetry [18–21]. However, these techniques are not applicable to researching the behaviour of the clusters on the single particle scale, because the signal is too weak [22]. Scanning Transmission Electron Microscopy (STEM) has thus been widely used to observe the behaviour of individual clusters [23, 24]. Due to its strong chemical sensitivity [25], clusters with multiple components and a complex structure can be observed in situ by STEM [26–28]. The recently developed temperature-controlled sample holders [29] have made STEM, and especially aberration-corrected STEM (ac-STEM), a very strong tool for observing the melting behaviours of metal clusters [30].

Bimetallic alloy clusters are receiving significant attention in recent years, especially these that combine low-cost and precious metals, as to retain the catalytic activities of the latter at a reduced cost, or to exploit the synergistic effects of different metals that give rise to unique physical and chemical properties [31–35]. The melting behaviour of the core–shell clusters can be significantly different from the corresponding monometallic particles.

Earlier molecular dynamics (MD) studies on the melting of Cu-Ni [36] and Ag-Co [37] nanoclusters found structural transitions that indicate a two-step melting process. The latter work found a strong dependence of the melting behaviour on the shell thickness in the core/shell nanoclusters. Since then, MD simulations have been performed on various systems, including Au-Pt [38, 39], Pt-Pd [40], Au-Cu [41] and Ag-Cu [42]. Two-stage melting behaviour has been observed in these systems, and the melting points of the core and shell show dependence on the core–shell ratio and their structure. The existence of the shell enhances the thermal stability of the core due to the confinement (pressure) effect. The melting of the core is suppressed even if the melting point of the shell is lower than that of the core [38]. When the melting point of the shell is much higher, the core melting is suppressed, and the melting point can even increase to a temperature higher than the bulk melting point [39]. A two-step process was also reported for the melting of AgAu, PtPd and AuCu nanoclusters of all core@shell combinations [43].

The inverse process, the solidification of AgCo and AgNi bimetallic nanodroplets, was recently studied and phase segregation was observed as the droplets were cooled to room temperature, since Co and Ni areas solidified at higher temperatures than Ag areas [44]. Furthermore, segregation of the constituent elements in bimetallic nanoparticles at elevated temperatures has been reported both experimentally and theoretically, due to the different surface energies and diffusivities between the constituent metals [45–47].

The different melting properties of core and shell are not easy to observe in situ even using STEM, if contrast is low between the constituent elements, i.e. the metal atoms are of similar atomic number. Moreover, previous atomistic-level studies of the melting behaviour of bimetallic core–shell nanoparticles represent only limited studies of the effect of confinement, especially the shell thickness, on the melting behaviour.

We report an atomistic simulation study of the melting of Pb-Al core–shell clusters during continuous heating. A series of MD simulations of core–shell clusters with a fixed Pb_147_ core and varying Al shell thickness has been employed together with detailed structural analysis to provide insights into the melting behaviour. Unexpectedly, a unique type of behaviour has been observed, in which the core atoms escape from the ‘prison’ of the encapsulating shell and reverse segregate to cover the shell atoms after the core melts. The selection of Pb and Al was made to provide a system open to STEM analysis—the much lower atomic number of the Al shell should enable the Pb core to be imaged. Moreover, the much higher melting point of the shell might supress the melting of the core. Our results further showed that the escape temperature changes as the core melting point varies, when the shell thickness is varied.

## Methods

### Models

The choice of the materials in this work was based on their very different atomic size and bulk melting temperatures. The nanoclusters comprise a core made of a low melting point and high atomic number element (Pb: ${T}_{m}$ = 600.6 K, $Z$ = 82, ${m}_{a}$ = 207.2 u) and a shell made of a high melting point and low atomic number element (Al: ${T}_{m}$ = 933.5 K, $Z$ = 13, ${m}_{a}$ = 27 u). Both metals crystallise in the face-centered cubic (fcc) crystal structure.

Before starting the core–shell simulations, we checked the energies of Pb and Al elemental clusters of sizes from ~ 200 to ~ 2k atoms for the octahedral and the three magic-number (cuboctahedral, decahedral and icosahedral) structural motifs. The energetic ordering for Pb clusters was cuboctahedral < decahedral < icosahedral, while for Al clusters it was icosahedral < cuboctahedral < decahedral   (Additioanl file 1: Figure S1) . The octahedral clusters with less than ~ 500 atoms were in both cases higher in energy compared with the highest energy magic-number clusters for each metal (icosahedral for Pb and decahedral for Al). The cuboctahedral motif was chosen, since it ranked 1st and 2nd for the two metals. The choice of the cuboctahedral offers two additional benefits: its layer-by-layer structure enables the building of core–shell clusters, and its crystalline nature allows for the use of structure analysis algorithms for the identification of the melting temperature.

**Fig. 1:**
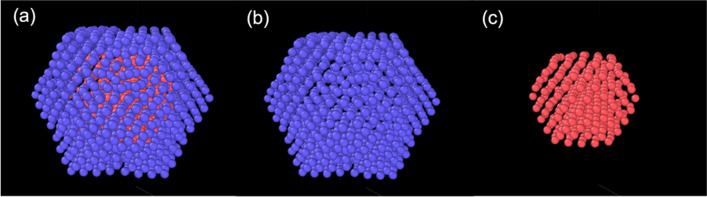
The structure of a cuboctahedral Pb_147_Al_776_ core–shell cluster after the initial relaxation. **a** The whole core–shell structure; Pb is marked in red and Al is marked in blue. **b** The shell part of the cluster. **c** The Pb_147_ core of the cluster

The following series of magic-number cuboctahedral Pb clusters was used: Pb_561_, Pb_923_, Pb_1415_, Pb_2058_, Pb_2869_. The core–shell structure was achieved by replacing outer shells of Pb atoms with Al while keeping a Pb_147_ cluster in the centre unchanged. Thus, the resulting initial configurations of the core–shell clusters were Pb_147_Al_414_, Pb_147_Al_776_, Pb_147_Al_1268_, Pb_147_Al_1911_ and Pb_147_Al_2722_ (2 to 6 layers of Al atoms surrounding the Pb_147_ core). A free Pb_147_ cluster was also simulated for comparison purposes. Figure 1 shows the structure of the Pb_147_Al_776_ cluster after the initial relaxation in (a), the Al shell of this cluster in (b) and the Pb_147_ core in (c). Mismatches and defects of the shell can be seen after the energy minimisation in Fig. 1a due to the large difference between the lattice constants of the core and shell atoms.

### Simulation details

All the simulations were carried out with LAMMPS Molecular Dynamics (MD) simulator [48], and the observation and analyses of the structural results were performed with OVITO [49]. An empirical many-body embedded atom model (EAM) potential [50] was employed to describe the interaction between atoms. The MD simulations were carried out in the canonical ensemble (NVT), and the Nose–Hoover thermostat was used to maintain the constant-temperature condition. Before the MD simulations, the initial clusters were fully relaxed. The integral calculation was done with the Verlet algorithm with a time step of 2 fs. As with all MD simulations, the detailed results are dependent on the initial conditions and run time. The temperature was set to increase from 0 to 1400 K, with a rate of 14 K/ns. The rate of the increase in the temperature was low enough to consider the system to be close to an equilibrium state during the simulation process. Each initial configuration was simulated five times to reduce the randomness in the results. The bulk melting points of Pb and Al were determined using NVT simulations of cubic boxes with periodic boundaries comprising ~ 10^3^ atoms in a fcc arrangement.

## Results and discussion

### The melting behaviour

In free clusters, surface melting precedes core melting as proposed by the liquid nucleation and growth (LNG) model of Couchman and Jesser [51]. In this model, a liquid shell is formed which gradually expands towards the core until the particle melts completely and suddenly when a critical radius is reached. This is not the case for core–shell particles, which demonstrate a more complex behaviour.

**Fig. 2:**
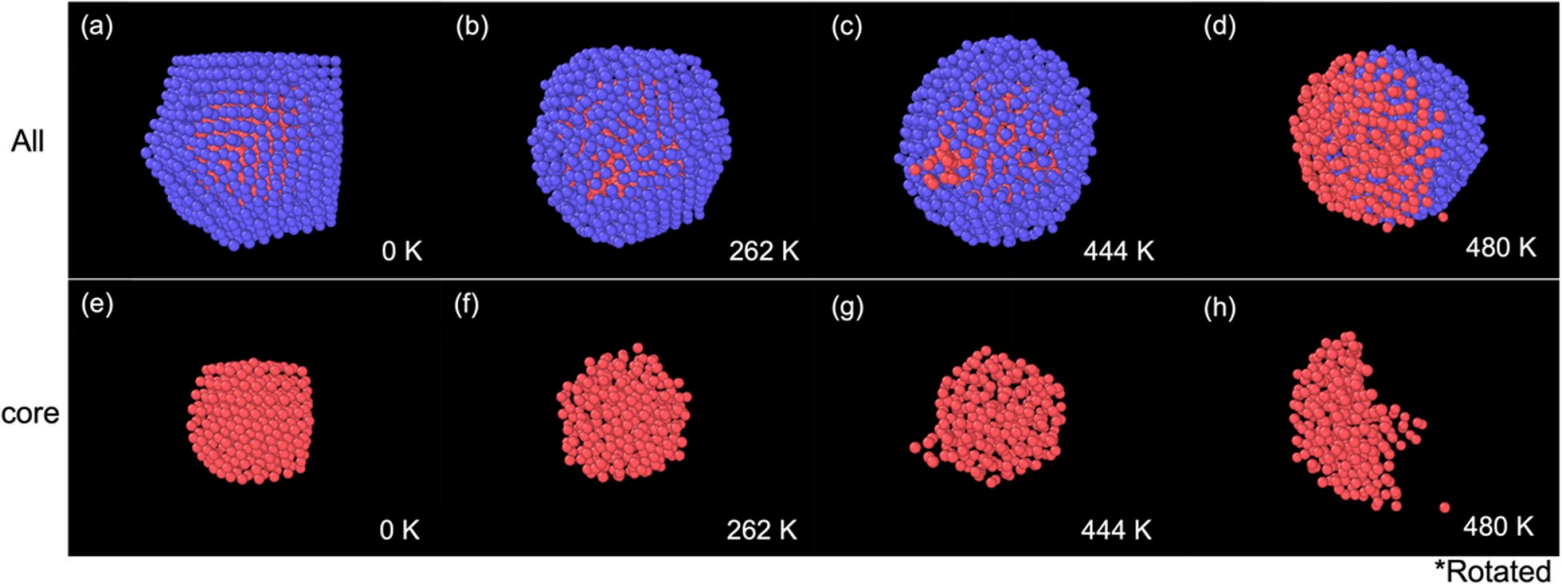
The melting behaviour of a Pb_147_Al_1268_ core–shell cluster. **a** The initial structure. **b** The core of the cluster is melted. **c** Core atoms begin to break out of the surface. **d** The core atoms have completely escaped. Panels **e**–**h** show the corresponding Pb core structures of panels (**a**–**d**). Panel (**h**) is rotated to show the resulting structure during the escape process of the core atoms

The melting process of a Pb_147_Al_1268_ core–shell cluster is shown in Fig. 2a–d. Figure 2e–h shows the corresponding core structure. The initial configuration after the relaxation is shown in Fig. 2a. The melting process can generally be divided into three phases. During the core melting phase, as the temperature increases, the core part starts to melt. Defects are randomly formed on the shell part due to the perturbation of the atoms, as shown in Fig. 2b. During the ‘prison-break’ phase, the core atoms eventually find a defect to break through and reach the cluster's surface, as shown in Fig. 2c. During the segregation phase, the core atoms gradually come out of the shell, as shown in Fig. 2d, and form a segregated nanocluster, otherwise known as a Janus nanocluster (a reference to the two-faced god of ancient Rome). The shell part melts during the ‘prison-break’ phase in most cases, apart from the smallest Pb_147_Al_414_ cluster, where it will melt before the melting of the core.

**Fig. 3:**
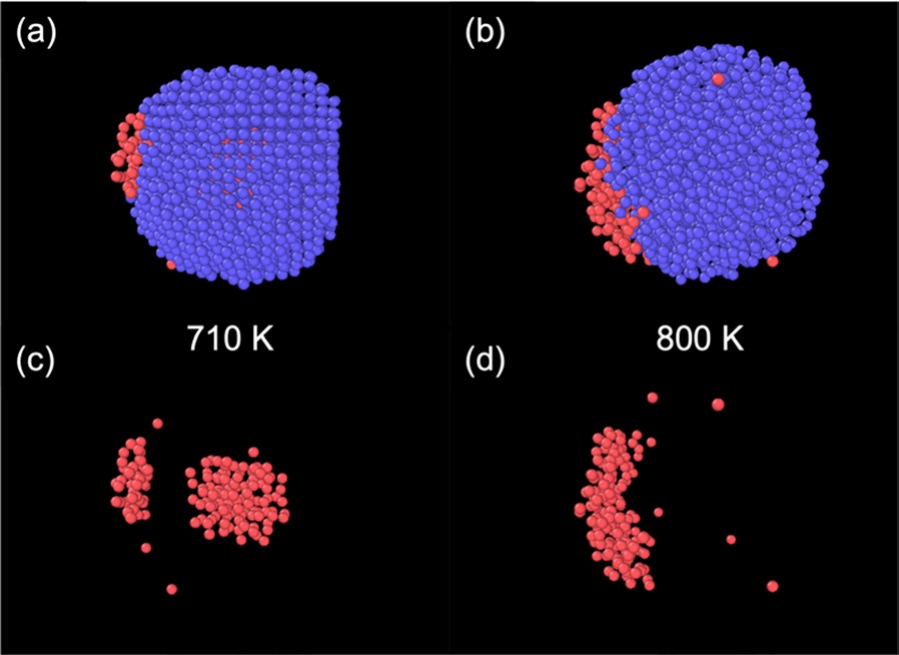
**a** The two-step break-out in a Pb_147_Al_1911_ nanocluster. Some of the core atoms are squeezed out, while the rest of the core remains inside the shell. **b** The core atoms will eventually break out at a higher temperature. Panels (**c**, **d**) show the core atoms corresponding to (**a**, **b**)

At a certain level of shell thickness (specifically, for Pb_147_Al_1268_ and Pb_147_Al_1911_; the later ones shown in Fig. 3), the escape will happen in more than one step. A part of the core is sometimes squeezed out of the Al shell, while the rest of the core remains inside the cluster (Fig. 3a). The remaining part in the centre can break out later at a higher temperature (Fig. 3b). The appearance of this multi-step prison break was observed in multiple simulations of the same structures, but no rule could be extracted concerning the number of atoms in the temporary remaining part.

Previous theoretical studies on segregation in nanoclusters focused on either neighbouring elements in the same period of the periodic table or elements in the same group of the periodic table [46], and they cannot be applied in our case. However, density functional theory (DFT) and MD studies on the segregation of Pb and Al in nano-crystalline and bulk materials showed that the segregation is governed by a combination of electronic effects and the size mismatch effect [52–54]. Clear grain boundaries between the Al and Pb phases were observed in these works, similar to our results for the Pb-Al clusters.

### Melting temperature and timing of the break-out event

**Fig. 4:**
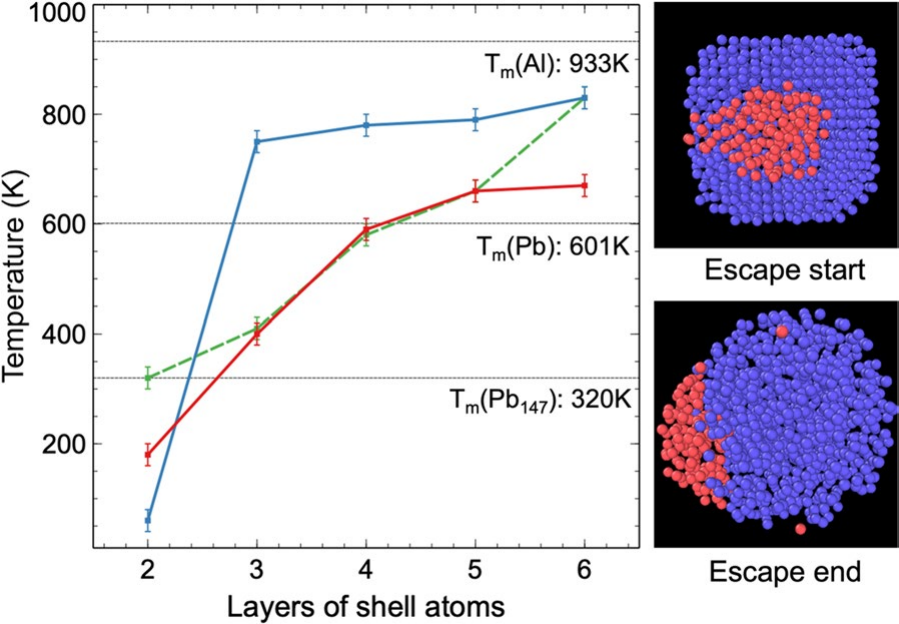
Melting and break-out event temperature of the Pb_147_Al_414~2722_ core–shell clusters (2 ~ 6 layers of shell atoms). The red line denotes the core melting temperatures and the blue line denoted the shell melting temperatures. The green dashed line shows the beginning of the core’s escape. The melting points of an elemental Pb_147_ nanocluster, bulk Pb and bulk Al using our simulation settings are marked with horizontal lines

The relationship between the melting point and the break-out event for different shell thicknesses (2 ~ 6 atom layers, Pb_147_Al_414~2722_) is shown in Fig. 4 and summarised in Table 1. The determination of the melting points is described in the following section concerned with the structural analysis of our models. The melting points of an elemental Pb_147_ cluster and bulk Pb and bulk Al were simulated using the same parameters that were used in our previous runs for comparison purposes. The melting point of the Pb_147_ core has a monotonic positive correlation with the thickness of the shell. The core of the Pb_147_Al_414_ cluster (2 shell layers) has a lower melting point than the pure Pb_147_ cluster, although it is covered by the shell. In the Pb_147_Al_1911_ and Pb_147_Al_2722_ clusters (5 and 6 shell layers, respectively), the melting point of the core is even higher than the melting point of bulk Pb. The shell melts at a higher temperature compared to the core, except in the case of Pb_147_Al_414_ (2 shell layers). The melting point of the shell has a monotonic positive correlation with the thickness as well.

**Table 1:** Melting and break-out temperatures of the Pb_147_Al_414~2722_ core–shell clusters (2 ~ 6 layers of shell atoms)

Cluster	Shell layers	Core melting temperature (K)	Shell melting temperature (K)	Escape start (K)	Escape end (K)
Pb_147_Al_414_	2	180	60	320	410
Pb_147_Al_776_	3	400	750	410	440
Pb_147_Al_1268_,	4	590	780	580	770
Pb_147_Al_1911_	5	660	790	660	780
Pb_147_Al_2722_	6	670	830	830	840

The temperature error is ± 10K

The beginning of the break-out occurs just after the core melts. Pb_147_Al_414_ is an exception: the break-out event happens at a higher temperature after the melting of the shell and core because of its relatively low total kinetic energy when the core is melted. Another exception is the largest Pb_147_Al_2722_ cluster (6 shell layers). The core atoms stay inside the cluster due to the stability of the very big shell until the shell is totally melted. The break-out process happens in two steps in clusters with 4 or 5 shell layers (Pb_147_Al_1268_ and Pb_147_Al_1911_). The first break-out happens just at the beginning of melting, when the whole structure is relatively stable, while the remaining Pb atoms in the core escape when the shell is completely melted.

**Fig. 5:**
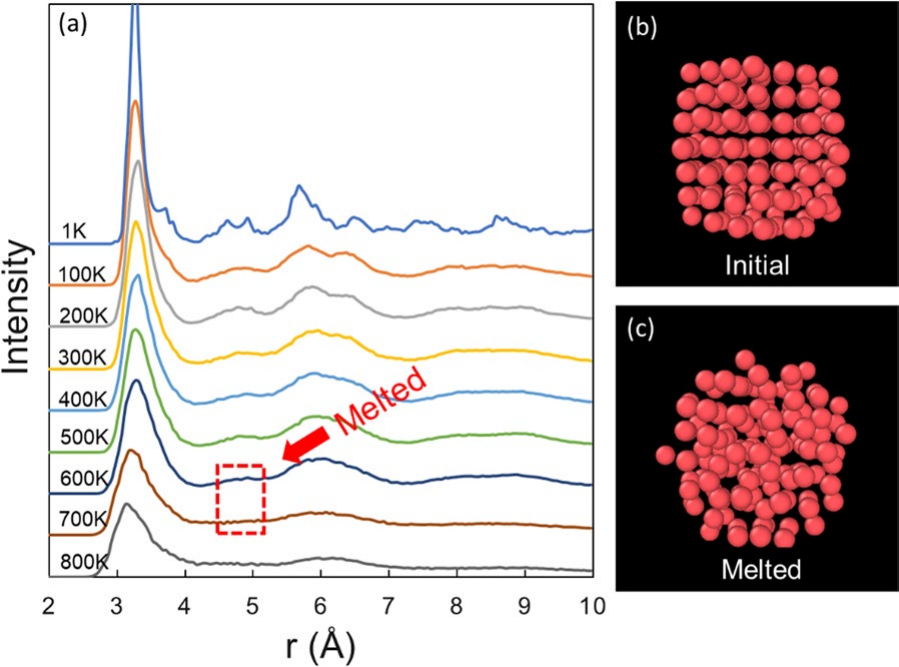
**a** Averaged pair distribution function (PDF) of the core atoms of the Pb_147_Al_2722_ core–shell cluster in different temperature ranges. **b** The initial core structure of the Pb_147_Al_2722_ cluster. **c** The structure of the core when the melting happens. It can be seen that the break-out event has not yet occurred when the core is melting

The melting point of the Pb_147_Al_414_ cluster is lower than that of the elemental Pb_147_ cluster; a fact that has not been observed in previous studies on the melting behaviour of core–shell clusters. Previous research shows that the melting point of a nanoparticle can be depressed by the insoluble impurities due to the perturbation of the lattice in the host clusters [55]. In the Pb_147_Al_414_ cluster, the mass ratio $\mathrm{Al}:\mathrm{Pb}$ is $0.36:1$. Al and Pb are insoluble, and defects can be seen on all the core structures (Fig. 1c), Fig. 5b, etc.). One can assume diffusing Al atoms as impurities in the Pb core to explain this melting point depression compared to the free cluster. Melting as a process initiates at defects and the Pb_147_Al_414_ cluster is abundant in defects.

In the Pb_147_Al_1911_ and Pb_147_Al_2722_ clusters (5 and 6 shell layers), the melting point of the core is higher than that of the free Pb_147_ nanocluster and even higher than that of bulk Pb. This result may be due to the pressure effect introduced by the increasing thickness of the Al shell, which has been previously mentioned in works on Al nanoparticles [56]. This showcases a new and interesting mechanism of manipulating the melting point of low-melting point metal droplets by confining them in a higher melting point shell.

The trigger for the break-out shifts from the melting of the core to the melting of the shell as the shell gets thicker. This is due to the fact that a thin defected shell has a lower melting point and is not robust enough to prevent the liquid state Pb atoms from getting out. However, as the shell gets bigger and more robust, the core needs to melt first in order to escape. The two-step break-out, which appears when 4 or 5 shell layers are present, is the intermediate scenario between these two conditions. When the shell is big enough, one might observe for a span of more than 100 K a liquid Pb droplet confined in a crystalline Al cage, before the shell melts and the core escapes.

### Structure analysis

The breaking out and segregation behaviour is crucially affected by the melting of the cluster. A pair distribution function (PDF) analysis was performed on the clusters to shed light onto their melting process. Previous research has shown the relationship between the averaged PDF and the melting behaviour of the clusters [57]. An averaged PDF within 50 frames (temperature range: 5 K, time range: 357 ps) has been applied to the core and shell atoms of the Pb-Al core–shell clusters, respectively. The averaged PDF of the core atoms of Pb_147_Al_2722_ at different temperatures is shown in Fig. 5a. The nearest neighbour distance ($\sigma \approx 2.5$ Å) is given by the occurrence of the first peak. As the temperature increases, the $\sqrt{2}\sigma$ peak (second peak) of the function begins to decrease and vanishes at the melting point. In Fig. 5a, the melting point of the Pb core is located from 600 K ~ 700 K. The accuracy of the melting point can be increased by repeatedly performing the averaged PDF analysis in this temperature range every 10 K. In this case, the extracted melting point of the core was $670\pm 10$ K. The initial and melted structures of the core are shown in Fig. 5b, c. The break-out has not yet happened, as shown in Fig. 5c. It is important to note that the break-out event always occurs after the melting of the core in all our simulations.

**Fig. 6:**
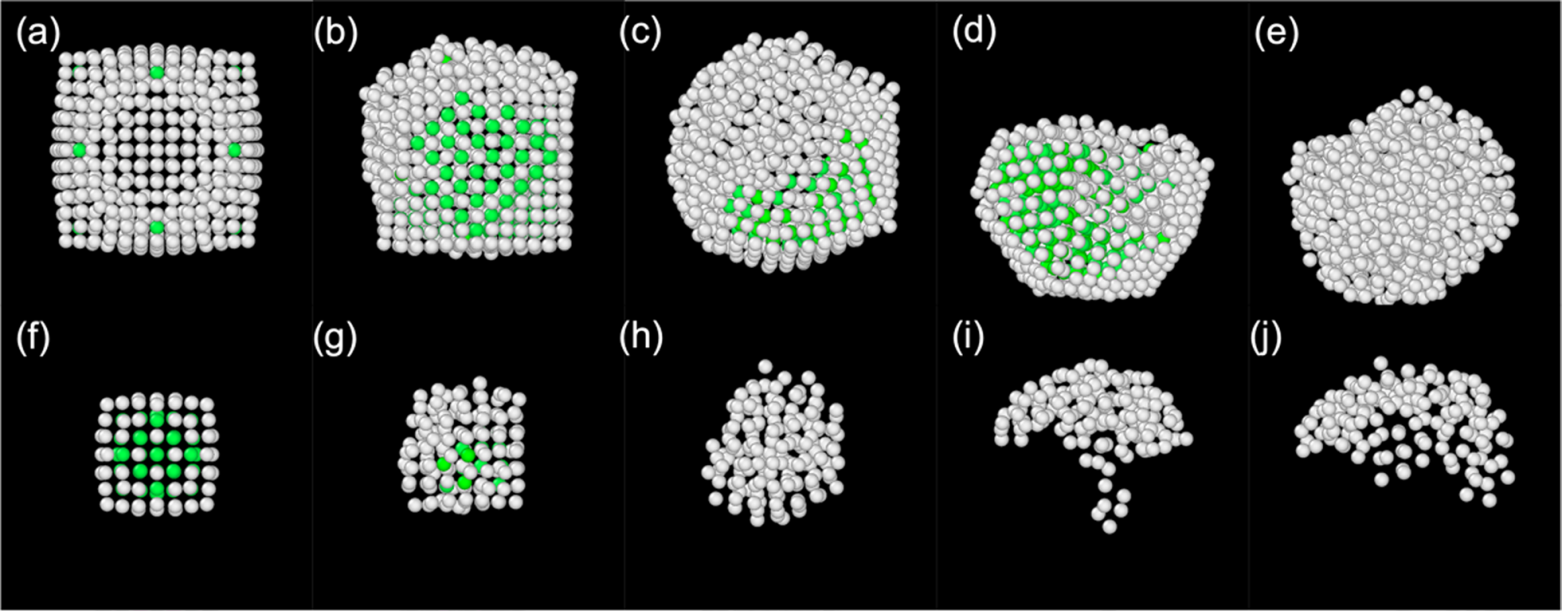
The adaptive common neighbor analysis (a-CNA) algorithm is used to study the melting process of a Pb_147_Al_776_ cluster. Αtoms identified as crystalline by the a-CNA are marked in green, atoms identified as non-crystalline in grey. The melting processes of the core (**f**–**j**) and shell (**a**–**e**) are shown separately. In the initial setting (**a**, **f**), the cluster is in a crystalline state. After a period of perturbation (**b**, **g**), the core melts (**c**, **h**) and breaks out of the shell (**d**, **i**). The shell maintains its crystalline structure during the break-out process. As the temperature increases (**e**, **j**), the shell eventually melts as well

Identifying the disappearance of the second peak can be challenging. A structure analysis algorithm can be employed to confirm the previously mentioned melting temperatures. Therefore, the adaptive common neighbour analysis (a-CNA) [58] was used to study the structural changes that occur during the melting process of core–shell clusters. Figure 6 shows the melting process of a Pb_147_Al_776_ cluster. In the initial state shown in Fig. 6a, f, the atoms after the outer layer are all recognised by the algorithm as crystalline, specifically of a face-centered cubic structure. The outer layer atoms, despite being in a clearly crystalline formation, are not recognised by the algorithm as such since they lack the required number of neighbours. After a period of perturbation, shown in Fig. 6b, g, the core of the cluster melts first (Fig. 6c, h). The break-out event then occurs almost immediately after the core melts. As shown in Fig. 6d, i, the shell remains crystalline during the entire break-out event. As the temperature increases (Fig. 6e, j), the shell eventually melts as well. In some cases, a transition to quasi-icosahedral local structures was observed in the Al shell by eye, which can be attributed to the fact that the icosahedral motif has lower energy than the cuboctahedral for Al clusters. However, as shown below, all the clusters remain largely crystalline up to the point where they suddenly melt. Clusters with thicker shells (Pb_147_Al_1268~2722_) behave differently, as previously described. The melting temperature results from the pair distribution and structure analyses were in very good agreement.

**Fig. 7:**
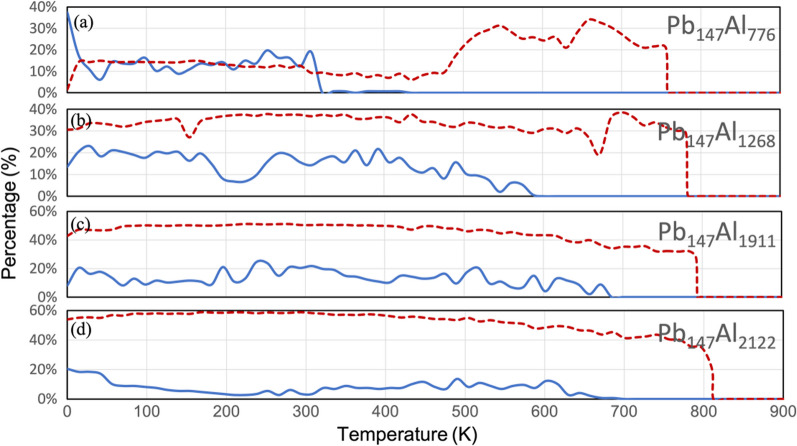
The proportions of crystalline atoms marked by a-CNA during the simulation, i.e. the crystalline Pb/the total Pb and the crystalline Al/the total Al. The blue solid lines represent the Pb core and the red dashed lines represent the Al shell. The data of Pb_147_Al_776~2122_ nanoclusters are shown in (**a**–**d**), separately. The core or the shell are considered melted when the percentage of crystalline atoms drops to 0

The proportions of crystalline atoms identified by a-CNA in core–shell clusters with different shell thicknesses during the entire simulation process are shown in Fig. 7. The fluctuations in the crystallinity of the core and the shell are generally within the range expected from elemental nanoclusters [30]. The data for Pb_147_Al_414_ is not shown because the number of atoms in the cluster that are marked as crystalline even at the beginning of the simulation is not sufficient for a proper analysis. This may be due to a combination of factors, including the very low melting point due to the smaller size, a higher proportion of surface atoms, and a more unstable structure due to defects introduced by shell atoms. The data for Pb_147_Al_776~2122_ are shown separately in (a)–(d).

According to the definition of melting, the number of crystalline atoms show a sudden decrease when melting occurs. This is more obvious in the shell atoms due to the larger number of atoms. Although there are large fluctuations in the data for the core, the melting temperature can still be accurately determined: after a certain point the atoms of interest are all recognised as amorphous. All the melting temperatures agree well with those from the PDF analysis. It can be observed that as the shell thickness of the core–shell clusters increases, the melting point of both the core and shell increases, with the increase in the core being more pronounced due to the stronger constraints of a thicker shell. Additionally, a sharp increase in the crystalline proportion of the shell can be observed at around 500 ~ K in the Pb_147_Al_776_ cluster. This is because after the break-out event, the cluster becomes a Janus nanoparticle, and the shell atoms reorganize into a structure with more bulk atoms that can be recognized by a-CNA.

**Fig. 8:**
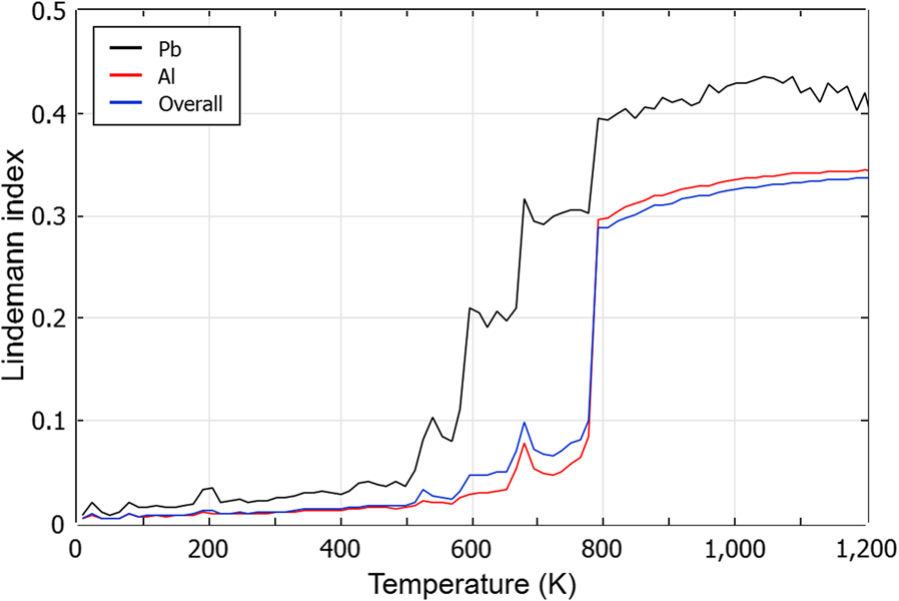
Lindemann index of the Pb_147_Al_1268_ cluster. The black line is Pb and the red line is Al. the blue line is the index for the whole cluster

Complementing this observation, the Lindemann index [59] offers a quantitative perspective on the melting transition of the core–shell cluster. Figure 8 illustrates the Lindemann index for the Pb_147_Al_1268_ cluster across varying temperatures. A stark increase around 780K can be observed in the Al shell (depicted by the red line). This sharp ascent signifies the shell's melting point. Conversely, the Pb core data (represented by the black line) showcases a more gradual climb, with a notable initial spike around 590K, pinpointing the core's melting onset. These observations dovetail precisely with the insights gleaned from our prior PDF and a-CNA analyses. Meanwhile, the overall index value of the cluster (blue line) is very close to the Al shell because the Al shell takes the majority of the atom number in the cluster.

## Conclusions

In summary, the melting process of Pb-Al core–shell clusters with different Al-shell thicknesses was studied using molecular dynamic simulations. The Pb core atoms break out of the Al shell and the nanoclusters end up in a segregated state after the melting of the clusters. The process may happen in two steps, in which case part of the core remains inside the cluster while the other part breaks out. The melting point of the Pb core can either be depressed or elevated by the presence of the Al shell, depending on the shell thickness. The thinner shell can be seen as insoluble impurities that introduce defects and mismatches to depress the melting point of the core, while the thicker shell elevates the melting point due to the introduction of the pressure effect. The timing of the break-out shifts from the melting of the core to the melting point of the shell as the shell gets thicker.

One can identify three distinct cases: i) when the core is loosely confined by a thin shell both the core’s and the shell’s melting temperatures are supressed and the shell melts before the core, ii) when the core is sufficiently constrained by several shell layers its melting temperature increases (compared to the corresponding free nanocluster of the same size) and it needs to melt in order to escape, and iii) when the shell is thick enough, the melted core will remain inside the crystalline cage until the shell melts and the core has the chance to escape. A comparison with Monte Carlo simulations for the same system under elevated temperatures might provide additional insight into the final structures.

Our research has provided new insight into the melting behaviour of the core–shell clusters. These results indicate that the stability and melting properties of core–shell clusters can be tuned by adjusting the thickness of the shell. We pinpoint melting point as yet another property that allows for tailoring in nanocluster science. Our work also provides a prediction and a reference for future experimental studies, especially STEM observation results.

### Supplementary Information

**Additional file 1: Figure S1. **The energy per atom of Pb (left) and Al (right) elemental clusters of sizes from ~ 200 to ~ 2k atoms for the three magic-number and for the octahedral structural motifs.

## Data Availability

The data that support the findings of this study are available upon reasonable request from the authors.

## References

[1] Kaden WE, Wu T, Kunkel WA, Anderson SL (2009). Electronic structure controls reactivity of size-selected Pd clusters adsorbed on TiO 2 surfaces. Science.

[2] Cheng N (2016). Platinum single-atom and cluster catalysis of the hydrogen evolution reaction. Nat Commun.

[3] Xie B (2022). Metal nanocluster-metal organic framework-polymer hybrid nanomaterials for improved hydrogen detection. Small.

[4] Scholl JA, Koh AL, Dionne JA (2012). Quantum plasmon resonances of individual metallic nanoparticles. Nature.

[5] Cao P (2020). Killing oral bacteria using metal–organic frameworks. Ind Eng Chem Res.

[6] Tyo EC, Vajda S (2015). Catalysis by clusters with precise numbers of atoms. Nat Nanotechnol.

[7] Wells DM, Rossi G, Ferrando R, Palmer RE (2015). Metastability of the atomic structures of size-selected gold nanoparticles. Nanoscale.

[8] Yin F, Wang ZW, Palmer RE (2011). Controlled formation of mass-selected Cu-Au core-shell cluster beams. J Am Chem Soc.

[9] Johnson, R. Atomic and molecular clusters. (CRC Press, 2003). doi:10.1201/9781482289305.

[10] Schmidt M, Kusche R, Kronmüller W, Von Issendorff B, Haberland H (1997). Experimental determination of the melting point and heat capacity for a free cluster of 139 sodium atoms. Phys Rev Lett.

[11] Lai SL, Guo JY, Petrova V, Ramanath G, Allen LH (1996). Size-dependent melting properties of small tin particles: nanocalorimetric measurements. Phys Rev Lett.

[12] Shvartsburg AA, Jarrold MF (2000). Solid clusters above the bulk melting point. Phys Rev Lett.

[13] Joshi K, Kanhere DG, Blundell SA (2002). Abnormally high melting temperature of the Sn10 cluster. Phys Rev B Condens Matter Mater Phys.

[14] Breaux GA, Benirschke RC, Sugai T, Kinnear BS, Jarrold MF (2003). Hot and solid gallium clusters: too small to melt. Phys Rev Lett.

[15] Haberland H (2005). Melting of sodium clusters: Where do the magic numbers come from?. Phys Rev Lett.

[16] Schmidt M, Kusche R, Von Issendorff B, Haberland H (1998). Irregular variations in the melting point of size-selected atomic clusters. Nature.

[17] Calvo F, Spiegelmann F (2000). Mechanisms of phase transitions in sodium clusters: from molecular to bulk behavior. J Chem Phys.

[18] Wu J (2022). Ultrafast atomic view of laser-induced melting and breathing motion of metallic liquid clusters with MeV ultrafast electron diffraction. Proc Natl Acad Sci USA.

[19] Kern J, Biesiadka J, Loll B, Saenger W, Zouni A (2007). Structure of the Mn4-Ca cluster as derived from X-ray diffraction. Photosynth Res.

[20] Bahena D (2013). STEM electron diffraction and high-resolution images used in the determination of the crystal structure of the Au144(SR)60 cluster. J Phys Chem Lett.

[21] Zhao B (2015). Phase transitions and nucleation mechanisms in metals studied by nanocalorimetry: a review. Thermochim Acta.

[22] Baletto F, Ferrando R (2005). Structural properties of nanoclusters: energetic, thermodynamic, and kinetic effects. Rev Mod Phys.

[23] Li ZY (2008). Three-dimensional atomic-scale structure of size-selected gold nanoclusters. Nature.

[24] Nelli D, Rossi G, Wang Z, Palmer RE, Ferrando R (2020). Structure and orientation effects in the coalescence of Au clusters. Nanoscale.

[25] Pennycook SJ (1989). Z-contrast stem for materials science. Ultramicroscopy.

[26] Liao TW (2018). Unravelling the nucleation mechanism of bimetallic nanoparticles with composition-tunable core-shell arrangement. Nanoscale.

[27] Nakamichi H, Yamada K, Sato K (2011). Sub-nanometre elemental analysis of Cu cluster in Fe-Cu-Ni alloy using aberration corrected STEM-EDS. J Microsc.

[28] Schnedlitz M (2019). Effects of the core location on the structural stability of Ni-Au core-shell nanoparticles. J Phys Chem C.

[29] Harutyunyan AR (2009). Preferential growth of single-walled carbon nanotubes with metallic conductivity. Science.

[30] Foster DM, Pavloudis T, Kioseoglou J, Palmer RE (2019). Atomic-resolution imaging of surface and core melting in individual size-selected Au nanoclusters on carbon. Nat Commun.

[31] Xiao BB, Zhu YF, Lang XY, Wen Z, Jiang Q (2014). Al 13 @Pt 42 core-shell cluster for oxygen reduction reaction. Sci Rep.

[32] Sun D (2015). Light-harvesting nanoparticle core-shell clusters with controllable optical output. ACS Nano.

[33] Tao F (2008). Reaction-driven restructuring of Rh-Pd and Pt-Pd core-shell nanoparticles. Science.

[34] Wanjala BN (2010). Nanoscale alloying, phase-segregation, and core-shell evolution of gold-platinum nanoparticles and their electrocatalytic effect on oxygen reduction reaction. Chem Mater.

[35] Serpell CJ, Cookson J, Ozkaya D, Beer PD (2011). Core@shell bimetallic nanoparticle synthesis via anion coordination. Nat Chem.

[36] Huang S-P, Balbuena PB (2002). Melting of bimetallic Cu−Ni nanoclusters. J Phys Chem B.

[37] Van Hoof T, Hou M (2005). Structural and thermodynamic properties of Ag-Co nanoclusters. Phys Rev B.

[38] Yang Z, Yang X, Xu Z (2008). Molecular dynamics simulation of the melting behavior of Pt-Au nanoparticles with core-shell structure. J Phys Chem C.

[39] Huang R, Wen YH, Shao GF, Sun SG (2013). Insight into the melting behavior of Au-Pt core-shell nanoparticles from atomistic simulations. J Phys Chem C.

[40] Huang R, Wen YH, Zhu ZZ, Sun SG (2012). Two-stage melting in core-shell nanoparticles: An atomic-scale perspective. J Phys Chem C.

[41] López MJ, Marcos PA, Alonso JA (1996). Structural and dynamical properties of Cu-Au bimetallic clusters. J Chem Phys.

[42] Grammatikopoulos P (2016). Kinetic trapping through coalescence and the formation of patterned Ag-Cu nanoparticles. Nanoscale.

[43] Nelli D, Mottet C, Ferrando R (2023). Interplay between interdiffusion and shape transformations in nanoalloys evolving from core–shell to intermixed structures. Faraday Discuss.

[44] Nelli D (2023). Two-steps versus one-step solidification pathways of binary metallic nanodroplets. ACS Nano.

[45] Wanjala BN, Luo J, Fang B, Mott D, Zhong CJ (2011). Gold-platinum nanoparticles: alloying and phase segregation. J Mater Chem.

[46] Reyes-Nava JA, Rodríguez-López JL, Pal U (2009). Generalizing segregation and chemical ordering in bimetallic nanoclusters through atomistic view points. Phys Rev B Condens Matter Mater Phys.

[47] Deng L, Hu W, Deng H, Xiao S (2010). Surface segregation and structural features of bimetallic au-Pt nanoparticles. J Phys Chem C.

[48] Thompson AP (2022). LAMMPS—a flexible simulation tool for particle-based materials modeling at the atomic, meso, and continuum scales. Comput Phys Commun.

[49] Stukowski A (2010). Visualization and analysis of atomistic simulation data with OVITO-the Open Visualization Tool. Model Simul Mat Sci Eng.

[50] Landa A (2000). Development of glue-type potentials for the Al-Pb system: phase diagram calculation. Acta Mater.

[51] Couchman PR, Jesser WA (1977). Thermodynamic theory of size dependence of melting temperature in metals. Nature.

[52] Jang S (2008). Influence of Pb segregation on the deformation of nanocrystalline Al: Insights from molecular simulations. Acta Mater.

[53] Purohit Y (2008). Atomistic modeling of the segregation of lead impurities to a grain boundary in an aluminum bicrystalline solid. Mater Sci Eng A.

[54] Zhang X (2010). Element segregation on the surfaces of pure aluminum foils. Appl Surf Sci.

[55] Hock C (2008). Melting-point depression by insoluble impurities: A finite size effect. Phys Rev Lett.

[56] Mei QS, Wang SC, Cong HT, Jin ZH, Lu K (2004). Determination of pressure effect on the melting point elevation of Al nanoparticles encapsulated in Al2O3 without epitaxial interface. Phys Rev B Condens Matter Mater Phys.

[57] Delgado-Callico L, Rossi K, Pinto-Miles R, Salzbrenner P, Baletto F (2021). A universal signature in the melting of metallic nanoparticles. Nanoscale.

[58] Stukowski A (2012). Structure identification methods for atomistic simulations of crystalline materials. Model Simul Mater Sci Eng.

[59] Lindemann FA (1984). Calculation of molecular Eigen-frequencies. Phys Z (West Germany).

