# Supplementary material for: Core atoms escape from the shell: reverse segregation of Pb–Al core–shell nanoclusters via nanoscale melting
Source: Discov Nano. 2023 Nov 17;18(1):143. doi: 10.1186/s11671-023-03924-3 (PMC10656412; doi:10.1186/s11671-023-03924-3)
Supplement: Supplementary file 1 — Additional file 1: Figure S1. The energy per atom of Pb (left) and Al (right) elemental clusters of sizes from ~ 200 to ~ 2k atoms for the three magic-number and for the octahedral structural motifs. [file 11671_2023_3924_MOESM1_ESM.docx]

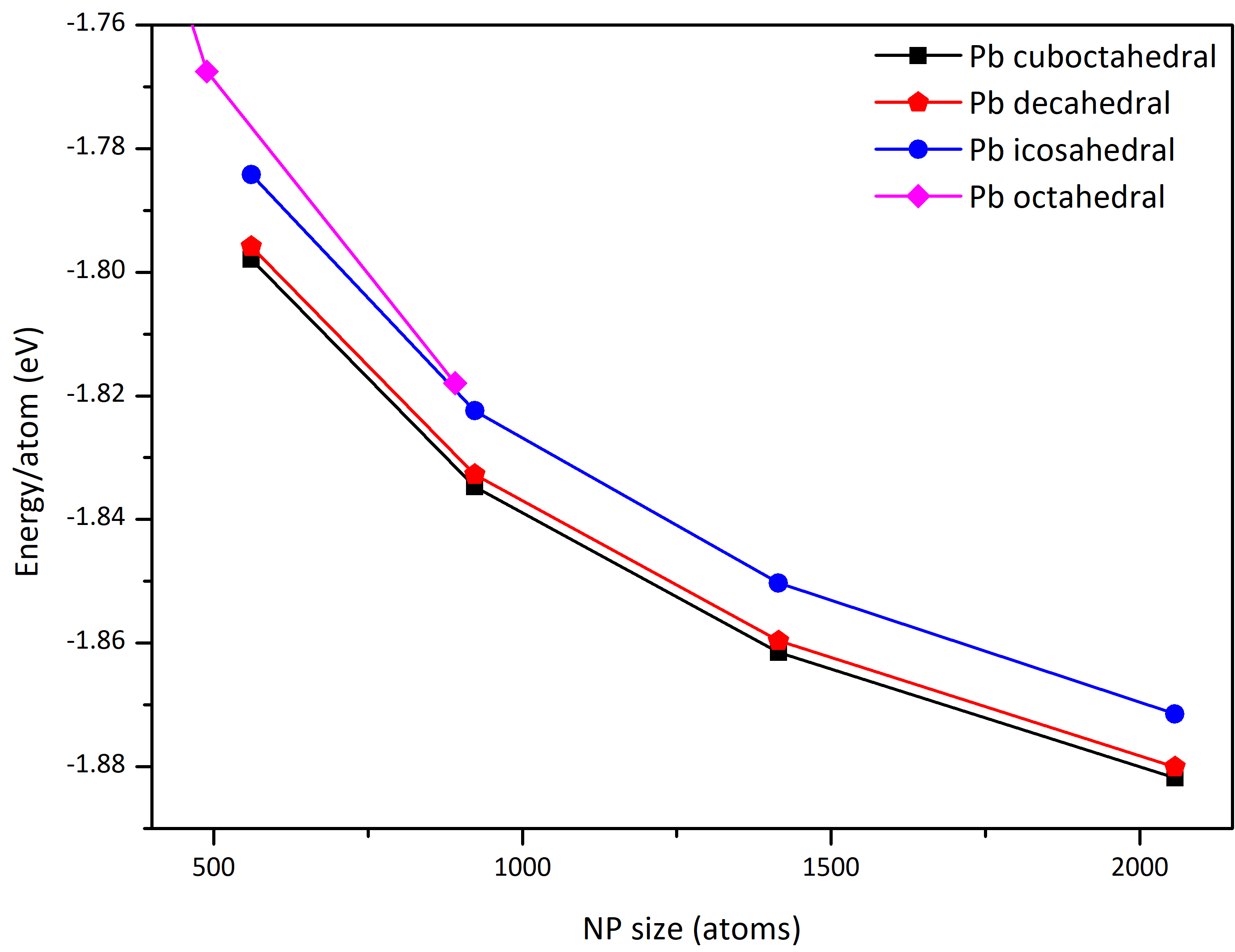

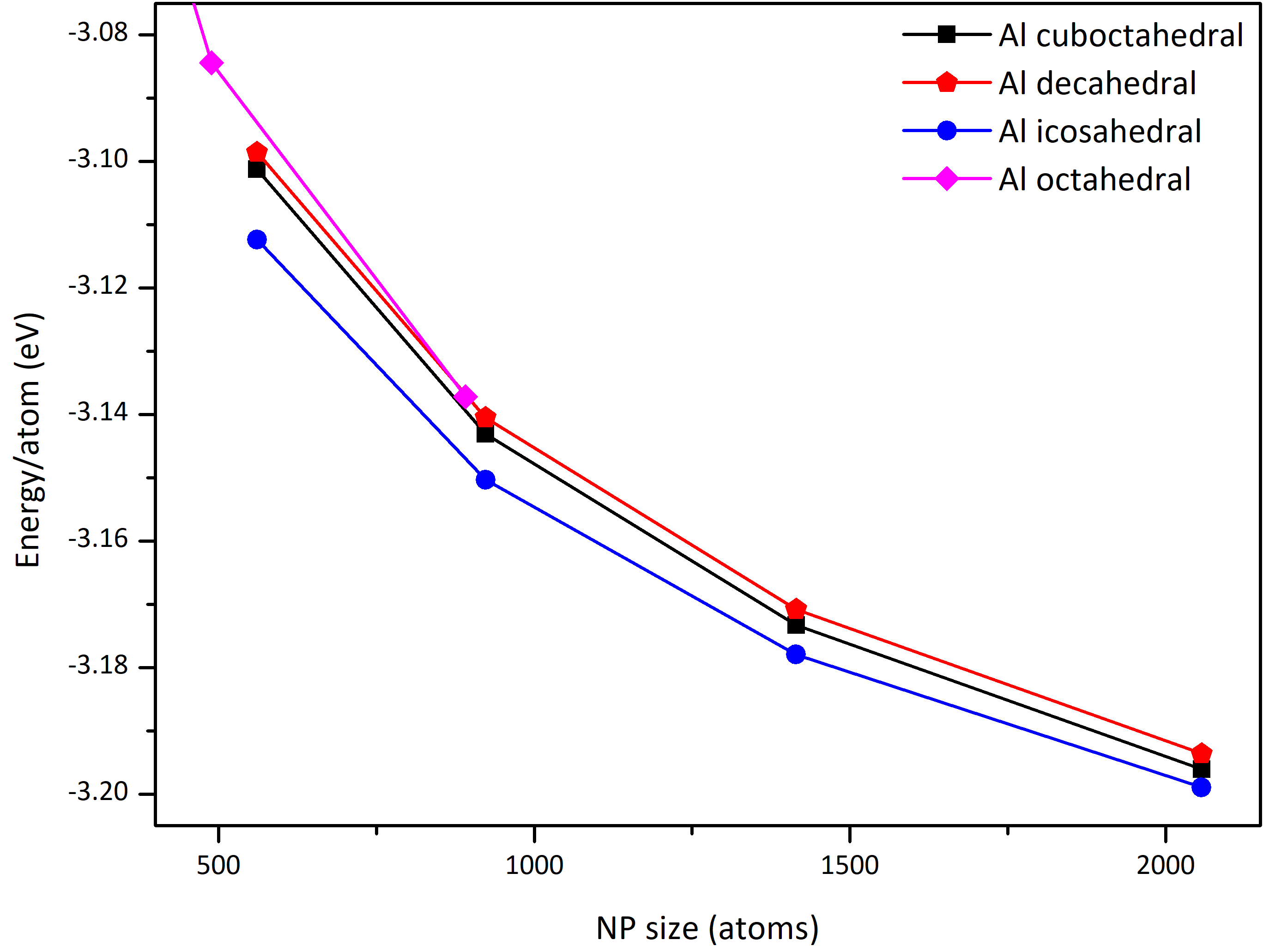


Figure S1. The energy per atom of Pb (left) and Al (right) elemental clusters of sizes from ~200 to ~2k atoms for the three magic-number and for the octahedral structural motifs.
